# Current and Future Lymphatic Imaging Modalities for Tumor Staging

**DOI:** 10.1155/2014/714674

**Published:** 2014-03-16

**Authors:** Ghulam Murtaza, Kuo Gao, Tiegang Liu, Imran Tariq, Ashif Sajjad, Muhammad Rouf Akram, Meiying Niu, Guokai Liu, Zahid Mehmood, Guihua Tian

**Affiliations:** ^1^Department of Pharmacy, COMSATS Institute of Information Technology, Abbottabad 22060, Pakistan; ^2^Beijing University of Chinese Medicine, 11 Bei San Huan Dong Lu, Chao Yang District, Beijing 100029, China; ^3^University College of Pharmacy, University of the Punjab, Lahore 54000, Pakistan; ^4^Institute of Biochemistry, University of Balochistan, Quetta 87300, Pakistan; ^5^Department of Pharmacy, University of Sargodha, Sargodha 40100, Pakistan; ^6^Beijing University of Chinese Medicine, Dongzhimen Hospital, Dong Cheng District, Beijing 100700, China

## Abstract

Tumor progression is supported by the lymphatic system which should be scanned efficiently for tumor staging as well as the enhanced therapeutic outcomes. Poor resolution and low sensitivity is a limitation of traditional lymphatic imaging modalities; thus new noninvasive approaches like nanocarriers, magnetic resonance imaging, positron-emission tomography, and quantum dots are advantageous. Some newer modalities, which are under development, and their potential uses will also be discussed in this review.

## 1. Introduction

The lymphatic system, also recognized as body drainage system, consists of capillaries, vessels, and nodes. Its function is to collect the lymph (rich in proteins and waste products) from the extracellular space within majority of the organs and put into the systemic circulation. It is also involved in the filtration of prospective immunogens from the extracellular space; thus the lymphatic system is considered as an important part of the immune system [1].

The conventional approaches used for the delivery of chemotherapeutic agents to the lymphatic system are not sufficiently promising and have resulted in the limited drug delivery to the lymphatic tissues. However, the concept of lymphangiogenesis (development of lymphatics and the growth of vessels) has enlightened the new role of lymphatics in cancer progression as well as its targeted therapy [2]. There is an active role of lymphatic system in the metastasis of cancer and distribution of infection. Once the tumor cells invade the regional lymph nodes, they rapidly spread to other parts of the body. However, the specific complex anatomy of lymphatics limits the localization of drugs in the lymphatic system. In new studies, specific markers carried by the tumor lymphatics have been revealed. These markers can be used to exclusively target the chemotherapeutic agents to the tumor lymphatic system [3]. In the literature, different drug delivery systems like microemulsions and nanoparticles, as well as various complimentary routes of administration like intramuscular, subcutaneous, oral, and intraperitoneal, have been narrated [1].

## 2. Literature Search Methodology

An extensive literature review in English was conducted, using electronic databases like EMBASE (1980–2012) and Medline (1966–2012). At the start, a test search was carried out using terms such as “lymphatics” and “scanning” simultaneously. Subsequently, different terms such as “polymers,” “quantum dots,” “tumor metastasis,” “lymphatic imaging,” and “sentinel lymph nodes” were combined with “lymphatics” and “scanning” for an advanced search. The literature evaluation was done by assessing the bibliographies of the selected publications demonstrating original researches for constructing an authentic review. The studies only in the English language were included in this study. Any duplication in data was excluded from the literature.

## 3. Results and Discussion

### 3.1. Lymphatic Imaging

For precise mapping of the drainage of solid tumors via lymph nodes, particularly the sentinel lymph node (SLN, the initial lymph node or cluster of nodes approached by metastasized cancer cells originated from solid tumor), a noteworthy curiosity has been observed in the development of imaging approaches. There is increased probability of presence of the metastasized tumor cells in the SLN comparison to other lymph nodes [19].

For cancer staging and therapy, the sentinel node has been focused in many clinical studies during the last 12 years [20]. In 1992, Morton et al. used vital dyes for the mapping of early stage melanoma. In their studies, they assumed that the sentinel node was principally responsible for the spreading of metastatic ailment [21]. Just after 2 years, Giuliano et al. employed the dyes in mapping the lymphatic metastasis in breast cancer [22]. In order to elaborate the possible role of the sentinel node in auxiliary metastasis in breast carcinoma, Turner et al. clearly showed the potential involvement of the SLN in the metastasis of breast carcinoma [23].

The investigation of the pathology of the lymph system has ever been problematic due to the specific complex anatomy of lymphatics. In comparison to bipedal lymphography (previous primarily used approach for the lymphatic imaging), the advance of noninvasive approaches like lymphoscintigraphy, ultrasound, computed tomography, positron emission tomography, magnetic resonance imaging, and optical imaging has facilitated the perfect assessment of lymph nodes [1, 23].

### 3.2. Lymphoscintigraphic Approach

It is a nuclear imaging approach involving the use of radionuclide. In lymphoscintigraphy, the subcutaneous injections are used for mapping the lymphatic drainage. The principally used radiolabelled agents are 99mTc-labeled human serum albumin, 99mTc-labeled dextran, or 99mTc-labeled sulfur colloid that exhibit the gamma ray emission [24–27].

The lymphoscintigraphy has two main limitations, that is, first, poor spatial resolution which does not allow assessment of the accurate anatomic site of sentinel node and, second, the short half-life of 99mTc (six hours) that requires the onsite generation potentials [1].

### 3.3. Ultrasonographic Approach

Ultrasonography (US) acts by preparing a map by creating the sound waves which get in touch with a surface or infiltrate the tissues and create an echo followed by the conversion of this echo into an image by the transducer [28]. Medical ultrasonography is carried out by four different mechanisms, that is, A-mode (a single transducer scans a line through the subject with the echoes plotted on the display as a function of intensity), B-mode (a group of transducers all together scan a plane through the subject that can be seen as a two-dimensional image on display), M-mode (a swift series of B-mode scans whose images go behind each other in series on the screen to assess range of motion), and D-mode (it involves the Doppler effect in the measurement and imaging the poignant constraints like blood flow). The B- and M-mode medical ultrasonographies are most frequently used for lymphatic imaging with respect to cancer [28].

The diagnosis of lymphatic problems in patients suffering from the cancer of head and neck is promisingly done using B-mode ultrasonography [29]. The transducers, having high-resolution as well as elevated frequency ranged between 5–20 MHz, produce optimal ultrasonographic results (90% accuracy rate) of the head and neck. The carcinoma of the tongue, salivary gland, and floor of the mouth can be detected by ultrasonography at both T1 and T2 staging [30]. In patients with post-thyroidectomy, ultrasonography is the most promising mapping approach to investigate recurrence of the thyroid cancer. The efficiency of the ultrasonography for detection of the metastatic lymph nodes can be augmented by coupling it with the detectable stimulated serum thyroglobulin [31]. The ultrasonography coupled with fine needle aspiration (FNA) has played an important role in the revealing of recurrence of thyroid cancer. This approach has exhibited 80–90% sensitivity in diagnosis of periodic disease in post-thyroidectomy patients with high levels of thyroglobulin [31]. For the detection and staging of pancreatic and rectal carcinomas, the development of endoscopic ultrasound (EUS) has become indispensable. The EUS-FNA has played an efficient role for the tissue diagnosis of pancreatic cancer [32]. A new meta-analysis has elaborated that EUS is an essential and perfect approach in the assessment of regional nodes in rectal cancer showing a pooled sensitivity and specificity of 73% and 76%, respectively [33].

In the future, there is possibility of the utilization of contrast enhanced ultrasound which augments the capability of ultrasound to assess the SLN as well as occult metastases through the use of a gray-scale sonographic contrast substance [18]. In addition, sonophoresis is another promising area of research where ultrasonography is used to augment the drug permeability through skin [4]. Another emerging ultrasound technology is the photoacoustic imaging which couples the ultrasound technology with laser [28].

### 3.4. Computer Tomographic Approach

X-ray computer tomographic (X-ray CT) modality is economical and thus a usually used diagnostic imaging tool. The mode of working of X-ray CT involves the visualization of difference in tissue density that gives image distinction by X-ray attenuation between electron-dense bone and soft tissues. To enhance the distinction between the cancerous and normal tissue, it is advantageous to use the X-ray contrast agents to increase the contrast of diseased tissue [34]. To know the tumor staging and lymphatic system, CT lymphography coupled with superb temporal and spatial resolution is generally employed. Some representative examples of CT distinction enhancers for the imaging of sentinel node include small hydrophilic iodinated organic moieties like iopamidol. The characteristic features of sentinel lymph node play an important role in its imaging. Suga et al. used iopamidol in CT-lymphography (CT-LG) to visualize the breast lymphatic system considering that breast tumors disseminate via the lymphatic pathways [35, 36]. They noticeably envisaged the direct linkage between the sentinel node and maximum CT attenuation of 269 ± 137 HU after subcutaneous injection of 0.5 and 1 mL of iopamidol to 10 female dogs [36]. In this study, no considerable side effects were observed. In next study in 17 human patients, they obtained correct imaging of the sentinel nodes after subcutaneous injection of 2 mL iopamidol into peritumoral and periareolar regions [37]. In 2008, Takahashi et al. administered subcutaneous injections to the breast cancer patients and accurately detected the sentinel nodes with CT-LG with an identification rate of 96% and 92% with dye guided procedures [38]. These results were also supported by Wu et al. [39].

Two main limitations of this approach are inadequate capability to get factual time images and strike of the ionizing radiation on the patient's body. However, the advancement in CT technology has significantly reduced the ionizing radiation employing some new CT technologies such as automatic exposure control and sinogram affirmed iterative reconstruction. These developments have helped to diminish the harmful outcomes and enhance the patient protection from CT radiation by using the adequately ample quantity of radiation to perform a CT examination. In this way, radiation contact is reserved as low as reasonably achievable (ALARA) [40], since CT is a digital technology that shows no apparent image quality-related benefit for amplified doses of CT radiation above a specific intensity. Rather, it merely deposits additional radiation into the body of patient [14, 41, 42]. The radiation contact to the patient can be considerably reduced by using the automatic exposure control (AEC) systems in current CT scanners. The usage of the AEC saves the radiation dose to a substantial degree and produces more uniform image noise in a scan [43]. In clinical settings, iterative reconstruction (IR) modality is another recently explored diagnostic approach that produces uniform low noise and high contrast images with good accuracy at significantly lesser radiation dose [44, 45]. Sinogram affirmed iterative reconstruction (SAFIRE) is the earliest raw-data-dependent IR that involves the use of algorithms for the improvement of image quality especially on exposure to low radiation dose. The SAFIRE-enabled reconstruction produced abdominal CT images devoid of decrease in diagnostic significance at 50% decreased radiation dose [46]. Later on, Kalra et al. reported good quality image of chest at reduced dose obtained by the SAFIRE [47]. In another study, Schabel et al. proposed potential radiation dose diminution of the novel SAFIRE modality in abdominal computed tomography angiography [48]. There are few studies that describe the role of IR modality for dose-saving in cardiac CT imaging [49, 50].

### 3.5. Positron-Emission Tomographic Approach

Positron-emission tomographic (PET) approach is a partially invasive scanning approach which is useful for detecting the lymphatic cancer through producing a three-dimensional image of metabolic processes in the body. Mechanistically, PET involves the detection of gamma ray pairs emitted from positron-emitting radionuclide as a tracer. These gamma rays are introduced into the body coupled with a bioactive substance such as F18-fluorodeoxyglucose (FDG) which is a glucose analogue. Through glucose transporters, body cells, specifically the inflammatory and cancer cells which exhibit higher rate of metabolism as compared to the normal cells, take up the FDG. Some newer agents like ((18)F)-2′-fluoro-2′-deoxy-1-beta-D-beta-arabinofuranosyl-5-ethyluracil ((18)F), (11C)-acetoacetate, and (11C)-choline are also being used for advanced detection of metastatic malignancies of breast and prostate [10, 51]. PET scanning is also used for the detection of lymphatics malignancies of gastrointestinal and lung [28]. Since local and metastatic diseases are efficiently detected by PET, PET coupled with CT (PET-CT) is promisingly used to detect gastrointestinal such as esophageal and gastric cancers [52]. There is increasing use of PET-CT scanning in lung and thorax cancers [53].

### 3.6. Magnetic Resonance Imaging

Magnetic resonance imaging (MRI) is one of the most useful noninvasive medical scanning modality; thus it is frequently used in clinical setup for mapping the tissue structure. In comparison to X-ray CT, MRI usually gives enhanced distinction between various soft tissues. MRI works through the mutual interaction of protons, magnetic field (for the perturbance of protons), and contrast agent (for altering longitudinal or transverse relaxation times) [1].

For improving contrast in MRI scanning of lymphatic pathways, various researchers have presented different contrast agents like iron oxide nanoparticles, gadolinium-diethylenetriamine pentaacetic acid (Gd-DTPA), and dendrimers and liposomes loaded with Gd(III) or iron oxides [54–56]. Nanoparticles loaded with iron oxide (superparamagnetic iron oxide nanoparticles, SPIONs) are advantageous because they are biocompatible, less toxic than optical scanning substances like quantum dots, and readily detectable at low quantities [57, 58]. For active targeting, antibodies, peptides, and proteins have been coupled with SPIONs. In an experimental murine model, Wunderbaldinger et al. developed dextran-SPION and used with contrast enhanced MRI for the detection of lymph node metastases [59]. Helbiech found ultrasmall superparamagnetic iron oxide (USPIO) particles (<50 nm diameter) as contrast substance for improving the capability of MRI to visualize the lymph nodes. For systemic lymph node scanning, USPIO nanoparticles possess long serum half-life [60, 61].

To detect lymph-node metastasis in 33 patients with prostate cancer through noninvasive elevated resolution MRI, Harisinghani et al. employed extremely lymphotropic superparamagnetic nanoparticles (2.6 mg of iron/kg of body weight) as contrast enhancers. The obtained MRI scans showed that the used MRI approach was superior in sensitivity (>90%) and diagnostic specificity (>97%) than conventional MRI [62]. In other studies [63–65], the authors used intravenous infusions of ferumoxtran-10 (2.6 mg of iron/kg of body weight) as contrast media and effectively detected the lymph node metastases in patients with prostate cancer. To detect the malignant lymph node in thirty patients with pelvic and urological cancers, Bellin et al. described high sensitivity (100%) and selectivity (80%) of USPIO showing no severe side effects [66]. In 9 patients with renal cell cancer, Guimaraes et al. also presented high sensitivity (100%) and specificity (95.7%) of method in lymph node scanning using ferumoxtran-10 [67].

Catzeddu et al. proved the axillary lymph node status as a potential aspect for staging and survivorship in breast cancer [68]. In some other studies [63–66], the authors employed immune and microscopic histological assessment approach after surgical dissection of the nodes; however this technique can be distressing and destructive resulting in severe problems. To know noninvasive staging of the axillary lymph nodes in 47 women suffering from new primary breast cancer, Murray et al. employed dynamic gadopentetate dimeglumine (Gd) enhanced MRI and found that sensitivity and specificity method were 100% and 56%, respectively [69]. Michel et al. used MRI coupled with USPIO and administered 100 mL infusion (2.6 mg of iron/kg of body weight) in saline to 20 patients for studying the preoperative evaluation of breast axillary lymph nodes and observed no severe side effects [70]. Kimura et al. utilized USPIO lymphography for the differentiation of normal and diseased nodes through enhancement patterns [71].

### 3.7. Optical Imaging

Optical imaging modality is advantageous over other techniques for lymphatic imaging because it is cost effective, noninvasive, and of greater speed and needs no exposure of any radiation and improved sensitivity and shows high resolution at shallow depths. In literature, different studies are reported for the optical imaging of the sentinel lymph node. However, this review describes the application of quantum dots (QDs) that are fluorescent semiconductor nanocrystals having distinctive electrical and optical features [72]. Their size ranges between 1–100 nm. QDs possess many excellent characteristics for example, 10–100 times more brightness than many dyes, quantum yield equal to approximately unity in comparison to the organic dyes and fluorescent proteins, extensive absorption properties, a comparatively lengthy fluorescence life time, a thin line width in emission spectra, continuous emission maxima, and insignificant photolightning. The absorption and emission properties of QDs depend upon its size and composition which is controlled by various synthetic approaches. In addition, the polymeric surface functionalization of QDs assists in targeting and avoiding from macrophage clearance [73, 74] which make QDs suitable for biosensing, cell labeling, nucleic acid assessment, and* in vivo* fluorescence scanning [75, 76].

An important application of QDs is to identify the lymph node pathways. The small size of QDs is responsible for its endocytosis by cells especially by the macrophages in lymph node localizing the QDs in sentinel lymph node facilitating for fluorescence scanning. If QDs do not localize in lymphatics away from sentinel node, and thus may not be promising for scanning the distal lymphatic drainage. Ballou et al. in 2004 coated four QDs with various surface coating agents for their localization. According to results, the QDs remained fluorescent for at least 4 months* in vivo* [77]. For mapping the SLN, Ballou et al. reported the movement of quantum dots with different charged surface groups injected into murine tumor models [9]. The investigators prepared dual coat QDs; first coating consisted of an amphiphilic polymer containing a large number of surface carboxy groups, while polyethylene glycols containing terminal carboxy, methoxy, or amino functional groups constituted the second layer resulting in the formation of QDs with no negative and positive charges. Thus the produced dual coat QDs contained cores as 800 nm emitting ZnS-CdSe-CdTe and 655 nm emitting ZnS-CdSe. These QDs having different sizes (neutral (22.6), negative (30.4), and positive (41.2)) were then injected to mice with M21 melanoma and MH-15 teratocarcinoma for fluorescence scanning. The results exhibited that size and charge did not affect the migration of QDs in the lymph nodes [9].

Based on the emission spectra, Kobayashi injected same-sized five different types of multiple QDs (Cd-Se with emission peaks at 565, 605, and 655 nm and CD-Te with emission peaks at 705 and 800 nm) to get multispectrum scanning of lymphatic pathways (Table 1) and concurrently observed five different lymphatic pathways which elaborated this modality better than other approaches like X-ray lymphangiography and MR-LG or radionuclide scintigraphy by which multiple lymphatic pathways cannot be tested all together [78, 79]. Bhang et al. used hyaluronic acid-QD conjugates (size range of 50–120 nm) to scan lymphatic drainage using an easy electrostatic coupling approach [16]. Robe et al. administered 20 *μ*L subcutaneous injection of QDs solution with a core of Cd/Se/ZnS emitting about 655 nm leading to the localization of QDs in the axillary lymph nodes in healthy nude mice. The produced scans showed a maximum concentration (2.42% of the administered dose) assessed after 60 min [80].

The QD fluorescence combined with radiotracer scintigraphy or MRI can be used to better map the deep nodes as compared to single techniques alone. Besides many advantages of QDs* in vivo* imaging over other traditional approaches, the use of QDs also presents some disadvantages such as the use of heavy metals in the synthesis of QDs raising questions about their safety [1, 6, 58].

Sharma et al. has successfully applied a near-infrared (NIR) fluorophore through intradermal injections for quantifying the lymphatic drainage [81]. In addition, the distinct scanning of the cancer cells and lymphatic pathways is also possible through the use of specific fluorescent antibodies. Licha et al. used fluorophore-conjugated antibody through intravenous injection to the marginal lymph nodes [82]. The authors confirmed the specificity of this antibody through the analysis of organ distribution and microautoradiography, and then developed MECA-79 antibody-near-IR cyanine fluorescent dye (with fluorescence and absorption peaks at 771 nm and 750 nm, resp.) conjugate (product 1). Using a control antibody, another conjugate having similar features was developed (product 2). Both conjugates were then administered through intravenous injection into the tail vein at identical doses in nude mice followed by fluorescent imaging. After 10 min of injection, it was observed that product 1 attached particularly with the lymph nodes, while product 2 failed to accumulate in the lymph nodes. Then the animals were sacrificed after 10 min of injections, lymph nodes were crushed and the product was analyzed for fluorescence signal intensity. The nodes with product 1 exhibited higher fluorescence intensities those that of product 2, identifying MECA-79 antibody as a suitable scanning investigation of lymph nodes. However, the MECA-79 antibody did not home in cancer cells specifically. To tackle this problem, Sampath et al. formulated dual-labeled monoclonal antibody (trastuzumab which is a therapeutic agent against HER2-positive breast cancer) specific for the HER2 (human epidermal growth factor receptor) homing at the surface of up to 30% of breast cancer and injected intravenously to the nude rabbits [83]. It was observed that cancer-specific antibodies can target cancer cells after delivering through the intravenous route.

It has been studied that there is an association between the VEGF (vascular endothelial growth factor)-C expression, lymphatic metastasis, and lymphangiogenesis through the interaction of VEGF-C with two receptors, VEGFR-2 and VEGFR-3 [75, 76]. He et al. combined the fluorescence lymphangiography with VEGFR-2 and VEGFR-3 specific antibodies to study the influences of VEGF-C on the development, function, and morphology of lymphatic system [84]. In this project, melanoma cells and fibrosarcoma were firmly engineered for the overexpression of VEGF-C and transduced for the expression of green fluorescent protein (GFP). Fluorescence lymphangiography and blue dye were used to study the lymphatic pathways present in the ear of nude mice followed by the implantation of VEGF-C overexpressing melanoma cells and fibrosarcoma at ear-tip. As compared to mock-transduced control cancer cells, these cells exhibited increased lymphatic metastasis due to the VEGF-C-induced increase of peritumor lymphatic channels. After 10 days of tumor implantation, the authors showed a 200-time increase in tumor cell buildup in the lymph nodes in tumors overexpressing VEGF-C in comparison to the control tumors using the fluorescence scanning approach. After the blockage of VEGFR-3, there was a reduction in the lymphatic hyperplasia as well as the tumor cell delivery to the lymphatic system, but no effect was observed on the growth of tumor cells implanted in the lymph node, while there was successful inhibition of growth of primary tumors and lymph node metastases after the blockade of VEGFR-2 [85, 86].

In a previous study, the fluorophore-conjugated lymphatic-specific antibody LYVE-1 has been combined with the fluorescent-protein-labeled cancer cells for achieving* in vivo* color-coded scanning of cancer cell moving in lymph nodes and lymphatic ducts [87]. LYVE-1, a hyaluronic acid receptor, is specific for lymphatic endothelial cells. The lymphatic pathways are likely immunohistochemically stained using anti-LYVE-1-antibodies. In this context, anti-LYVE-1-antibodies-AlexaFluor 488 conjugates were developed for imaging the lymphatic system, and then injected into the inguinal lymph node. In contrast, the injection of control FITC-dextran or conjugated IgG to the inguinal lymph node exhibited greatly reduced durable signals. The fluorescence microscopy was used* ex vivo* to verify the specificity of anti-LYVE-1-antibody [1, 87].

### 3.8. New Modalities

In addition to the traditional, standard, and currently used scanning approaches like CT, MRI, PET, and ultrasound methods for mapping of the lymphatic cancer, the emerging, and newly evolved modalities like photoacoustic imaging and nanotechnology are replacing these traditional techniques [28].

### 3.9. Photoacoustic Imaging

Photoacoustic imaging (PAI) is a hybrid biomedical mapping approach which couples the sonography and laser pulses for the delivery of energy as short pulses of a nonionizing laser into the biological tissues. A part of this delivered energy converts into heat through thermoelastic expansion. It creates an ultrasonic emission (i.e., photoacoustic signal) detectible by the ultrasound transducer translating these signals into an image. The degree of the photoacoustic signal is proportional to the local energy accumulation and exhibits the optical absorption contrast specific to physiological system permitting the formation of 2D and 3D images [88]. Kimura et al. injected the indocyanine green and detected its accumulation in the SLN of rats using noninvasive PAI. Song et al. injected the methylene blue and detected its accumulation in the SLN of rats using noninvasive PAI demonstrating that the SLN with 20 mm depth in 3D mapping and 31 mm depth in 2D mapping were potentially recognized [17]. Staley et al. successfully used* in vivo* noninvasive PAI to detect the metastasis of melanoma in rat brain to detect the implantation spot and any metastatic phenomenon [15].

### 3.10. Nanotechnology

Nanotechnology has been used to develop the engineered multifunctional tools, having nanoscale dimensions identical to those of bulky biological molecules in the body, for the diagnostic and therapeutic objectives. Nanoparticles have been used as passive as well as active targeted drug delivery tool to accumulate the drug in tumor cells or at its surface: possibly due to the damaged lymphatic pathways and permeable angiogenic ducts in the region of tumors. In recent studies, tumor cell recognizing molecules like fluorophores or peptides are being conjugated with the nanoparticles to augment the cell-specific attachment as well as uptake. Many studies on cancer involve the use of quantum dot, dendrimers, inorganic nanoparticles, and stealth liposomes [13].

The biorecognition conjugates, like hyaluronan (HA, a nonsulfated polysaccharide), have played very important role in the mapping of cancer lymphatics to enhance therapeutic outcomes. Many studies on HA have elaborated HA as a perfect target in biomedical research due to its wide distribution in body especially in connective, epithelial, and neural tissues as well as in lymphatics. In addition, an increase in metastasis and cancer progression has been observed with increase in HA formation and uptake [89, 90]. Cai et al. carried out the imaging of lymphatics to study the localization of hyaluronan nanoconjugates coupled with fluorescent tag (like Texas Red) in sentinel nodes in mouse [7]. In cancer lymphatic scanning, quantum dots (QDs, also termed as nanocrystals) are also used. On receiving energy (like ultraviolet light) from some external sources, the flushing of QDs is observed depending upon the number of atoms in QDs. Kobayashi et al. carried out the lymphatic imaging of melanoma cells labeled with QDs and dendrimers as a macromolecular contrast media after injecting into mice using* in vivo* multichannel fluorescence microscopy. Due to use of combination of QDs and dendrimer, an enhanced recognition of metastatic cancer cells within the lymphatic system was observed [12]. Ballou et al. successfully detected the SLN in teratocarcinoma and melanoma using fluorescent microscopy after injecting fluorescent-labeled QDs to mice. In addition, nanotechnology based QDs are being combined with standard scanning approaches like PET of MRI for noninvasive sentinel lymph node mapping for melanomas and breast cancer [91]. To treat some malignancy, inorganic nanoparticles have been coupled with magnetic resonance modality to develop magnetic nanoparticle for targeted delivery to cancer with the help of a magnetic field. Balivada et al. injected magnetic nanoparticles to mice with melanoma subjecting to an irregular magnetic field employing a magnetic resonance scanner showing an excellent antitumor activity as well as an edge over the existing state of the art modalities [8, 28].

Scintigraphy is an excellent modality used to develop and assess the liposome labeled with gamma-emitting radionuclide through two-dimensional imaging after intravenous injection of this swiftly excreted radioactive substance to the patient. Lymphoscintigraphy, a quite new technology in nuclear medicine mapping, is used to generate the two-dimensional scintigrams of lymphatics after administering a radioactive substance through oral, intravenous, or pulmonary route [5]. After travelling through lymph channels, the radioactive substance is taken up by the lymphatic system. During this course, gamma rays generated from the radioactive substance are identified by a PET scanner or gamma camera. This tool is generally employed for identifying the SLN and diagnosing the disease like lymphedema and lymphoma [92–95]. Using scintigraphic mapping, Pillips et al. successfully employed technetium-99m (99mTc) labeled liposomes for the assessment of viral infection, fractured bones, and delivery of liposomal product [96]. For synchronized distribution and excretion of isosulfan (pyrogen-free blue dye having low molecular weight), Plut et al. successfully prepared a formulation kit containing radioactive blue liposomal formulation: a combination of isosulfan and 99mTc labeled liposomes [97]. Fujimoto et al. employed long-circulating palmityl-D-glucuronide liposomes to formulate gadolinium-diethylenetriamine pentaacetic acid labeled liposomes which were successfully used to detect lymph nodes (popliteal as well as retroperitoneal) after injecting it subcutaneously into the hind feet of rabbits. The potential accumulation of liposomes in the lymphatic system was probably attributable to the macrophagal entrapment of liposomes [98–100].

The main problem of liposomes is their rapid clearance after injection. In order to develop long-circulating liposomes and their accumulation in diseased tissues and the SLN, Zavaleta et al. employed avidin/biotin-liposome systems in a rodent grafted with ovarian cancer demonstrating significantly enhanced uptake of liposomes by lymphatics after two hours of injection [101]. For the determination of tissue biodistribution of 99mTc-labeled liposomal formulations and the most useful injection site in rats, Medina et al. employed a modified avdin/99mTc-bluebiotin-liposomal formulation for targeting the mediastinal node demonstrating that the lymph node targeting effect did not depend upon the cavity in which the formulation was injected [102, 103].

## 4. Conclusion

An important role of lymphatic system has been observed in tumor progression. For cancer staging, new noninvasive lymphatic imaging modalities are being investigated eliminating the patient risks. Although all lymphatic imaging approaches involve the application of general tissue imaging methods, imaging agents developed exclusively for the lymphatic system will increase the efficiency of existing tools. During the last twenty years, there is an extensive research in lymphatic imaging, particularly cancer lymphatic imaging. In this context, new approaches include ultrasonography, PET imaging, MRI, nanoparticle detection, fluorescence, and photoacoustic imaging. The novel modalities in combination with comparatively traditional approaches have guided towards new developments in diagnostic as well as therapeutic scanning of lymphatic involvement in tumor progression. These scanning modalities can be useful in the targeted drug delivery and thus improved therapeutic outcomes.

## Figures and Tables

**Table 1:** Various research modalities.

Modality	Product	Type of cancer	Application	Reference
Ultrasound	Sonophoresis	Breast cancer	Therapeutic	Husseini and Pitt, 2009 [4]
Nanotechnology	Nanoconjugate drug	Breast cancer	Therapeutic	Cai et al., 2011 [5]
Nanotechnology	Stealth liposome	Breast cancer	Therapeutic	Qiang et al., 2009 [6]
MRI	Combine with ultrasound	Prostate cancer	Therapeutic	Nune et al., 2011 [1]
Nanotechnology	Nanoconjugate drug	Breast cancer	Therapeutic	Cai et al., 2010 [7]
Nanotechnology	Iron oxide	Melanoma	Therapeutic	Balivada et al., 2010 [8]
Nanotechnology	Quantum dot	Melanoma	Diagnostic	Ballou et al., 2007 [9]
PET	New tracer	Breast cancer	Diagnostic	Authier et al., 2008 [10]
PET	New tracer	Prostate cancer	Diagnostic	Mease, 2010 [11]
Nanotechnology	Quantum dot	Melanoma	Diagnostic	Kobayashi et al., 2009 [12]
Ultrasound	Contrast enhanced	Breast cancer	Diagnostic	Wang and Thanou, 2010 [13]
PET	New tracer	Melanoma	Diagnostic	Cody et al., 2004 [14]
Ultrasound	Photoacoustic imaging	Melanoma	Diagnostic	Staley et al., 2010 [15]
Ultrasound	Photoacoustic imaging	Breast cancer	Diagnostic	Bhang et al., 2009 [16]
Ultrasound	Photoacoustic imaging	Breast cancer	Diagnostic	Song et al., 2008 [17]
Ultrasound	Contrast enhanced	Breast cancer	Diagnostic	Weskott, 2008 [18]
